# A quantitative assessment of the parameters of the role of receptionists in modern primary care using the work design framework

**DOI:** 10.1186/s12875-020-01204-y

**Published:** 2020-07-10

**Authors:** Michael Burrows, Nicola Gale, Sheila Greenfield, Ian Litchfield

**Affiliations:** 1 Institute of Applied Health Research, College of Medical and Dental Sciences, University of Birmingham, Edgbaston, Birmingham, B15 2TT UK; 2 Present Address: School of Psychological, Social and Behavioural Sciences, Faculty of Health & Life Sciences, Coventry University, Priory St., Coventry, CV1 5FB Birmingham, UK; 3 School of Social Policy, HSMC Park House, University of Birmingham, Birmingham, UK

**Keywords:** Primary care, Health service delivery, Quantitative research

## Abstract

**Background:**

Amidst increased pressures on General Practice across England, the receptionist continues to fulfil key administrative and clinically related tasks. The need for more robust support for these key personnel to ensure they stay focussed and motivated is apparent, however, to be effective a more systematic understanding of the parameters of their work is required. Here we present a valuable insight into the tasks they fulfil, their relationship with colleagues and their organisation and their attitudes and behaviour at work collectively defined as their ‘work design’.

**Methods:**

Our aim was to quantitatively assess the various characteristics of receptionists in primary care in England using the validated Work Design Questionnaire (WDQ) a 21 point validated questionnaire, divided into four categories: task, knowledge and social characteristics and work context with a series of sub-categories within each, disseminated online and as a postal questionnaire to 100 practices nationally.

**Results:**

Seventy participants completed the WDQ, 54 online and 16 using the postal questionnaire with the response rate for the latter being 3.1%. The WDQ suggested receptionists experience high levels of task variety, task significance and of information processing and knowledge demands, confirming the high cognitive load placed on receptionists by performing numerous yet significant tasks. Perhaps in relation to these substantial responsibilities a reliance on colleagues for support and feedback to help negotiate this workload was reported.

**Conclusion:**

The evidence of our survey suggests that the role of modern GP receptionists requires an array of skills to accommodate various administrative, communicative, problem solving, and decision-making duties. There are ways in which the role might be better supported for example devising ways to separate complex tasks to avoid the errors involved with high cognitive load, providing informal feedback, and perhaps most importantly developing training programmes.

## Background

Over the last 15 years, general practice has experienced a profound increase in workload as the population ages and the complexity of care increases [1–4]. Demand has reached unprecedented levels [2, 5] and the primary care landscape is changing [6–8]. As a result, staff are now delivering care in a far more complex and dynamic environment with implications for clinical and non-clinical members of the primary care team. Amongst the most visible of these are receptionists who not only undertake an array of administrative duties [9, 10] but also fulfil clinically related tasks such as triaging patients, reporting results or administering screening [11–19] often without adequate training [10]. The failure of receptionists to successfully fulfil these responsibilities has potentially serious implications for patient outcomes and safety [15, 20–22].

The need for more robust support for these key personnel to ensure they stay focussed and motivated is apparent, but to be effective a more systematic understanding of the parameters of their work is required. This includes the tasks they fulfil, their relationship with colleagues and their organisation, and their attitudes and behaviour at work. This concept of understanding how the nature of work can reflect how well it is performed was first introduced by Herzberg [23] who described how jobs could be enriched and managed to foster responsibility and growth in competence. Building on this, the concept of job characteristics theory described how people would perform at their best when they were internally motivated to do so as opposed to the promise of some external reward or the threat of supervisory attention [24]. By its nature the design of an individual’s work shapes the contribution made to the organisation and offers an understanding of the experiences and behaviours of employees [25]. This ‘work design’ is a critical component of human resource management that when understood and optimised improves job satisfaction, the quality, safety and efficiency of the work, [26, 27] and has positive impacts on performance, absenteeism and turnover [28, 29]. In understanding work design and supporting its improvement the validated work design questionnaire (WDQ) [26], has proved a valuable tool producing benefits in a range of industries including information technology [30], nursing [31], and policing [32].

Whilst the most visible member of the practice team, the receptionist’s role has largely been overlooked and to date there has been no detailed exploration of the ‘work design’ of GP receptionists; especially important in the context of the changing landscape of primary care. This study marks the first time that an England wide survey of GP receptionists aimed to understand the extent of their current role and importantly how we can help them remain motivated, productive and effective within a system of high demand and limited resource. Additionally, this study also marks the first use of the WDQ with this occupational group.

## Methods

### Study design

The study was designed as a large scale survey study of the job design of receptionists in England, utilising an existing validated questionnaire, the WDQ [26] (See supplementary material 1).

### Research instrument

**Fig. 1:**
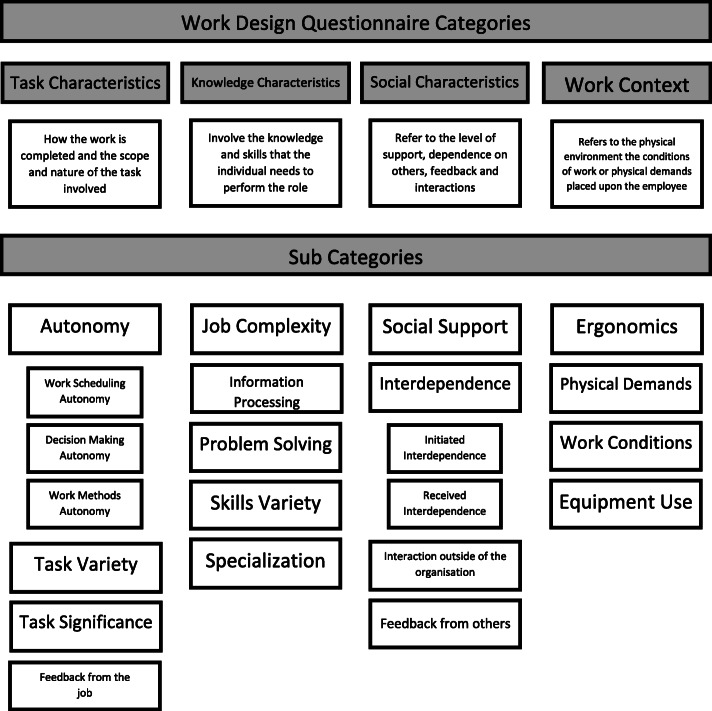
Work Design Questionnaire, Categories and Sub Categories

The WDQ [26] is a validated measure of work characteristics. It consists of a 21 point scale, divided into four groups each with sub-categories, responses to which are coded on a 5 point Likert Scale; from strongly disagree to strongly agree (Fig. 1). In addition, demographic details were collected for each participant including age, gender, disability, and ethnicity.

### Recruitment

Receptionists are difficult to access as there is no overall list for practices in England; therefore, multiple recruitment methods were employed. These included disseminating the link to the online questionnaire via Clinical Commissioning Groups in England, Health Education England, Association of Medical Secretaries, Practice Managers, Administrators and Receptionists and GP surgeries working with the University of Birmingham. Bristol Online Survey hosted the survey and the link directed the respondent to an information page, consent was required. In addition, as most practices have more than one receptionist, 500 postal questionnaires were sent to 100 randomly selected GP practices across England between September 2016 and September 2017.

### Sampling

All GP receptionists in England were eligible to participate. There were no exclusion criteria beyond job role. In 2014 (the most recent year for which there was data) there were 93,037 administrative and clerical staff in primary care, 67% of the primary care workforce [33]. Employing a 95% confidence interval and a margin of error of .5 a sample of 384 was required.

### Analysis

Following standard procedures for analysis of the WDQ [26], the respondent’s scores were added together for each of the subscales, a mean was drawn, presented as a percentage of the total possible score. Responses were then categorised as low (score less than 50% of the total score), moderate (scores between 50 and 75% of the total score) and high (above 75% of the total score) for each subscale.

## Results

Seventy receptionists completed the questionnaire, 16 postal questionnaires (3.1% response rate) and 54 online questionnaires. Sixty-nine (99%) were female, over half (56%) were aged 40 and over, and nearly half (49%) had been in post for longer than 5 years. These data are summarised in Table 1.

**Table 1:** Participant characteristics

Demographics							
*Gender Identity (%)*							
Female (%)	Male (%)						
69 (99)	1(1)						
*Age Range years (%)*							
18–28	30–39	40–49	50–59	60+			
15 (21)	16(22)	11(16)	21(30)	21(30)	7(10)		
*Level of Education (%)*							
No Qualifications	GCSE/CSE	Further Education	A Levels	Bachelors Degree	Post-Grad. Qualification		
3 (4)	27(39)	19 (27)	12 (17)	7 (10)	2 (3)		
*Marital Status (%)*							
Single	Living with partner	Married/civil partnership					
26 (37.7)	9 (13)	35 (49.3)					
*Disability (%)*							
Yes	No						
2 (2.9)	68 (97.1)						
*Sexual Orientation (%)*^a^							
Heterosexual	Gay woman/Lesbian	Gay Man	Bisexual	Other			
65 (96)	1 (1)	0	2 (3)	0			
*Religious Belief (%)*^a^							
No Religion	Christian	Muslim	Other				
31 (45.5)	35 (51.5)	1 (1.5)	1 (1.5)				
*Ethnic Background (%)*							
White	Pakistani	Other					
68 (97)	1 (1.5)	1 (1.5)					
**Occupational Characteristics**							
*Time in post (%)*^b^							
0–5 Years	6–10 Years	11–15 Years	16–20 Years	21 Years +			
35 (51)	16 (23)	10 (14)	4 (6)	4 (6)			
*Respondents Practice Size (%)*^b^							
Small	Medium	Large					
4 (6)	38 (55)	27 (39)					
**Geographical range**							
*Region (%)*^c^							
West Midlands	South	South West	East Anglia	North West	North East	East Midlands	South East
30 (45)	9 (14)	6 (9)	9 (14)	5 (8)	3 (4)	2 (3)	2 (3)

^a^completed by 68/70 correspondents

^b^ completed by 69/70 correspondents

^c^ completed by 66/70 correspondents

The results from the WDQ are presented below where we describe the key findings in each of the four categories, with the means and percentages given for each sub-category.

### Task characteristics

**Fig. 2:**
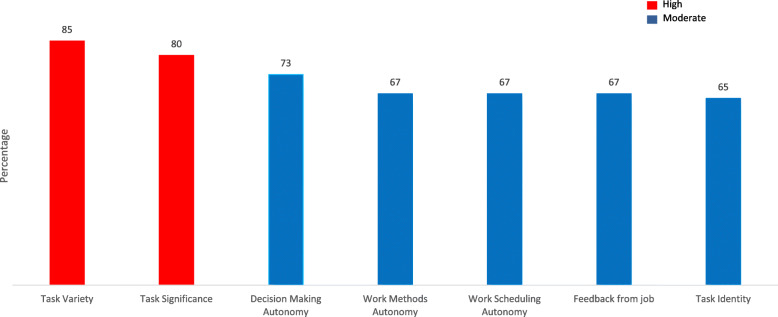
Task Characteristics Subscales, percentage of total score

Receptionists reported moderate levels of autonomy across the three subsets of work scheduling, decision making and work methods; decision making autonomy scored the highest (Mean score [m] = 3.62, 73%). Both task variety (M = 4.25, 85%) and significance (M = 4.03, 85%) were high. Task identity relating to whether an individual undertakes a single overall task or contributes to a smaller aspect of a larger service was moderate (M = 3.21, 65%). Feedback from the job relates to the extent that the role itself provides ‘direct and clear information’ on the effectiveness of their performance [26] was scored as moderate by receptionists (M = 3.25, 67%). These results are summarised in Fig. 2.

### Knowledge characteristics

**Fig. 3:**
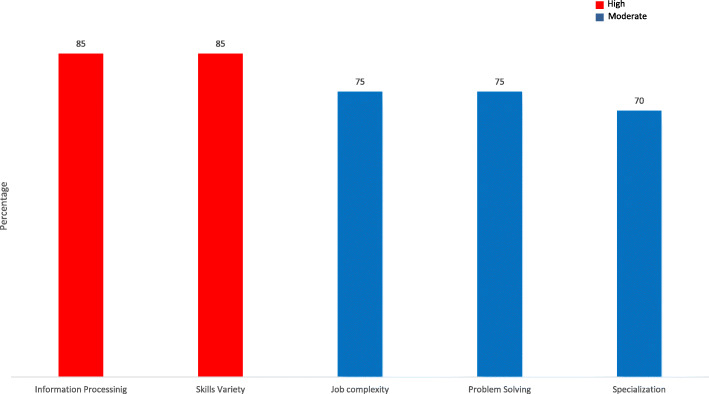
Knowledge Characteristics Subscales, percentage of total score

Knowledge characteristics include job complexity, the amount and type of information an individual must process to perform their role, the problem solving ability required, the variety of skills and the degree of specialisation required. Receptionists reported moderate complexity (M = 3.81, 75%) however informational processing demands were classified as high (M = 3.81, 75%). The need to develop original solutions and ideas was classed as moderate, bordering on high (M = 3.74, 75%). Skills variety was classed as high (M = 4.16, 85%). Reflecting the degree to which the role requires a wide variety of skills the need for specialized or specific knowledge was scored as moderate by those we surveyed (M = 3.43, 70%). These results are summarised in Fig. 3.

### Social characteristics

The social characteristics of a role relate to various social or interpersonal aspects of the job and the degree of support, advice and assistance (needed and received) in the workplace and was classed as high (M = 3.99, 80%).

**Fig. 4:**
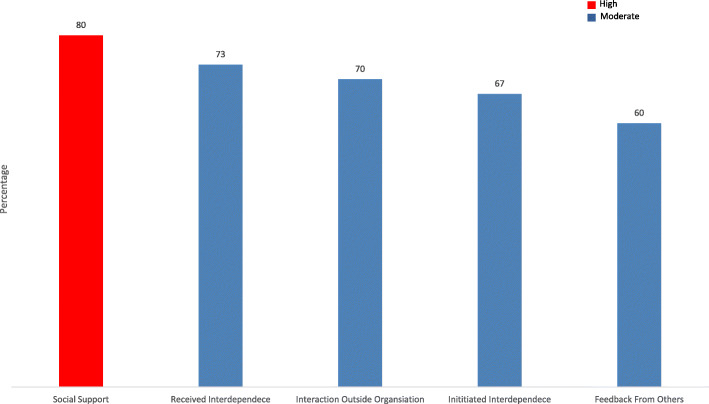
Social Characteristics Subscales, percentage of total score

Interdependence was divided into either initiated independence, referring to the extent one job flows into others or received independence the extent that the one role is affected by work from other jobs and both were classed as moderate (M = 3.30, 67%) and (M = 3.66, 73%). Receptionists scored the level of interaction with external agencies as moderate (M = 3.41, 73%) as they did feedback from their colleagues (M = 3.11, 60%). These results are summarised in Fig. 4.

### Work context

**Fig. 5:**
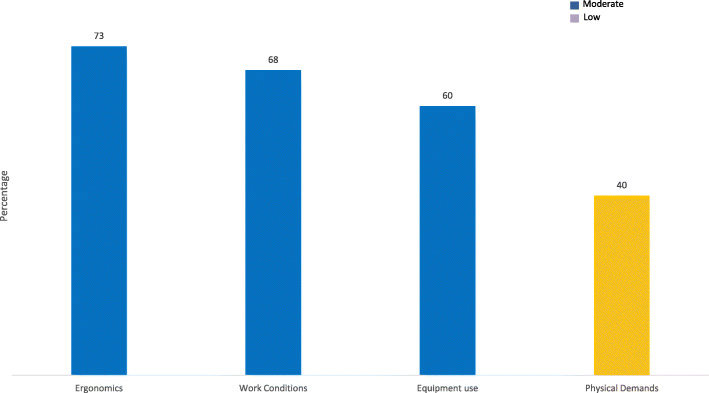
Work Context Subscales, percentage of total score (moderate scores in blue, low in yellow)

This covers the environment of the organisation in which the individual works and the physical demands placed on the employee in undertaking their roles. Receptionists scored the ergonomic value of their role as moderate (M = 3.51, 73%), the physical activity and effort required as low (M = 1.96. 40%) and the variety and complexity of the equipment needed as moderate (M = 3.01, 60%). Overall the working conditions which includes factors such as the existence of health hazards, cleanliness, noise were described as moderate (M = 3.43, 68%). These results are summarised in Fig. 5

.

## Discussion

### Summary

We used Hackman and Oldham’s theory of work design [28] to help us understand how the characteristics of a receptionist’s roles can resonate psychologically in terms of the meaningfulness of work, the level of responsibility assumed and the outcomes of their work. These criteria are fundamental to intrinsic motivation, and how successful their work has been, enabling them to learn from mistakes and connect emotionally to the result of their actions.

Our participants reported a high level of autonomy and variety in the work they do though were relatively uncertain as to the success of their individual contribution. They were required to process a high level of information and employ a wide variety of skills yet did not regularly receive feedback from their colleagues. The ergonomic and physical impact of their work was low. Below we describe these findings in more detail within each of the four domains of the WDQ; Task characteristics, Knowledge characteristics, Social Characteristics, and Work Context.

### Strengths and limitations

The survey was conducted amongst a number of GP practices and primary care environments across England [34] and the WDQ provided the first quantitative insight into the parameters of the role of receptionists, highlighting key aspects of their work and suggesting areas where additional support may prove beneficial. However we do not claim our results are generalizable, as though the demographic characteristics of receptionists in our group reflect those of previous studies [10, 35, 36]; our sample size is smaller than preferred and so our findings do not necessarily reflect those of every receptionist and general practice. Unfortunately the recruitment of a broader sample of receptionists was hindered by the lack of a centralised list of reception staff in England, which is perhaps a contributory factor as to why they remain a seemingly hard to reach research population [37].

### Comparison with existing literature

#### Task characteristics

Increasingly, modern surgeries are multi-disciplinary teams consisting of clinical and non-clinical staff each undertaking a range of inter-related tasks to successfully deliver care [38–41]. As such the work the receptionist undertakes is varied [9–11, 42–45] and straddles both clinical and non-clinical responsibilities [9–11, 14, 16–19, 43, 46–51]. In doing so the receptionist juggles multiple sources of information from patients, colleagues, and external agencies often with competing demands on attention; for example booking patients into the practice while simultaneously taking phone calls [17, 52]. High variety can be rewarding [26, 27] but can also lead to an overtaxed and underperforming workforce [26, 27].

In other environments such as aviation, issues of competing demands and multitasking have been tackled by introducing the idea of a ‘sterile cockpit’ which prohibits extraneous activities such as non-essential communication and reading non-essential materials during the critical phases of the flight [53]. Cognitive processing is undertaken serially and so multi-tasking is effectively “task-switching” between multiple tasks and so attention is shared sequentially [54]. This process slows down work and errors are more likely directly after the ‘switch’ has occurred [54, 55].

The implications of excessive cognitive load are especially important in healthcare where demand is high, information often incomplete and time constrained [56–58]. Distractions, interruptions, and external extraneous stimuli disrupt attention and can lead to error [56, 57]. Conversely, interruptions can be beneficial, offering information sharing needed for task completion [59], an alternate perspective, increasing positive affect [60] and when tasks are routine, distractions can speed information processing without concomitant negative effects on accuracy [59, 61]. For reception work, separating tasks may reduce the likelihood of error in complex tasks, for example separating greeting patients and answering the telephone into discrete roles may help to reduce error by minimising the interruptions encountered when undertaking these roles simultaneously. Similarly, complex work with potentially serious implications for patient safety such as repeat prescribing would benefit from being undertaken as a separate activity to reduce the cognitive load of multitasking [54, 55, 62].

#### Knowledge characteristics

The receptionist undertakes a number of roles that at times require specialised knowledge from triage [15, 20, 21], to repeat prescribing [21, 22]. However, no formal qualifications are required [10, 15] and much of the training that exists is provided in-house, from existing reception staff [36, 42, 63, 64] and viewed by receptionists as inadequate [10, 42, 63, 64]. Barriers to improving this training including time constraints, and a lack of funding and relevant courses [65]. Recently this training shortfall has been acknowledged and in 2017 Health Education England, established a £45 million fund to support training in two discrete roles, managing medical correspondence and active care navigation [66] though its effect on quality, safety and staff is as yet unknown.

#### Social characteristics

Social support in the workplace helps underpin well-being [67, 68] and psychological and behavioural functioning [69] in a range of jobs and environments, including policing [70] hospitality [71] and healthcare [69, 72]. Our sample described the level of feedback as ‘moderate’ yet receptionists have previously described how important it is to their well-being and job satisfaction [10, 42]. Though systematic mechanisms for providing feedback to receptionists exist, such as annual performance reviews and appraisals, [73] the time constrained and high pressured atmosphere of modern general practice precludes other avenues for providing the type of social support that might improve well-being [74]. This social connection also helps engender in reception staff a grasp of the outcomes of the work they complete. In other environments understanding the implications of their actions can help staff increase motivation and enable mistakes to be observed constructively [28] and could also be used to provide a framework for receptionists to monitor and improve performance.

#### Work context

Work environment directly affects an employee’s ability to perform their role [25–29]. Receptionists are some of the most visible members of the practice team [16], their front of house position can bring them into contact with difficult or aggressive patients [75] or leave them feeling dissociated from the rest of the primary care team [42, 43]. Although their location in the practice is unlikely to change, some of the negative effects might be mitigated by the opportunity for receptionists to share their experiences with supervisors and colleagues [76, 77].

The receptionist regularly uses information technology (IT) to manage patient data and service delivery. These clinical software systems are used to manage patient records, prescribing, test results and appointment bookings as well as facilitating communication from GPs to receptionists [78]. Despite their pivotal role a recent survey found that 12% of receptionists received no training in their use [65] despite evidence of errors linked to their misuse [15, 21]. A sociotechnical perspective is one theory that has previously been adopted to improve the fit between individual and IT system and can be used to ensure the design of healthcare IT is informed by the context of the individual and their work environment [79].

## Conclusions

Though receptionists continue to fulfil many of their traditional roles, the demands and complexity of modern primary care means they are being placed under increasing pressure to do so safely and effectively. Reducing cognitive load, improving training and feedback, and ensuring that IT systems harmonize with personnel and work practices can only help. Further research should aim to validate the findings from this study with a larger sufficiently powered sample. In addition, it would be helpful to design future studies in ways that are powered to detect differences between regions and types/size of practice. Meanwhile it is important that the issues identified by this study with respect to the receptionist’s role within existing systems and processes are acknowledged and addressed as soon as possible.

## Supplementary information

**Additional file 1.**

## Data Availability

All data generated or analysed during this study are included in this published article.
