# Supplementary material for: A quantitative assessment of the parameters of the role of receptionists in modern primary care using the work design framework
Source: BMC Fam Pract. 2020 Jul 10;21:138. doi: 10.1186/s12875-020-01204-y (PMC7353756; doi:10.1186/s12875-020-01204-y)
Supplement: Supplementary file 1 — Additional file 1. [file 12875_2020_1204_MOESM1_ESM.docx]

**Work Design Questionnaire (1)**

The questions in this part concern characteristic of your job itself, this section will explore autonomy, knowledge required for the job, social characteristics and finally the work context.

Using the scale below, please indicate the extent to which you agree with each statement. Remember to think only about your job itself, rather than your reactions to the job.

1 = Strongly Disagree 2 = Disagree 3 = Neither Agree nor Disagree 4 = Agree 5 = Strongly Agree

Please respond as accurately and honestly as possible. There are no right or wrong responses. For each question, choose the response option on the scale that best corresponds to your opinion. This section of the questionnaire should take less than 15 minutes to complete.

**Part 1 - Autonomy**

**Work Scheduling Autonomy**

1. The job allows me to make my own decisions about how to schedule my work.

| 1  Strongly Disagree | 2 Disagree | 3  Neither Agree nor Disagree | 4 Agree | 5  Strongly Agree |
| --- | --- | --- | --- | --- |
|  |  |  |  |  |

2. The job allows me to decide on the order in which things are done on the job.

| 1  Strongly Disagree | 2 Disagree | 3  Neither Agree nor Disagree | 4 Agree | 5  Strongly Agree |
| --- | --- | --- | --- | --- |
|  |  |  |  |  |

3. The job allows me to plan how I do my work.

| 1  Strongly Disagree | 2 Disagree | 3  Neither Agree nor Disagree | 4 Agree | 5  Strongly Agree |
| --- | --- | --- | --- | --- |
|  |  |  |  |  |

Decision-Making Autonomy

1. The job gives me a chance to use my personal initiative or judgment in carrying out the work.

| 1  Strongly Disagree | 2 Disagree | 3  Neither Agree nor Disagree | 4 Agree | 5  Strongly Agree |
| --- | --- | --- | --- | --- |
|  |  |  |  |  |

2. The job allows me to make a lot of decisions on my own.

| 1  Strongly Disagree | 2 Disagree | 3  Neither Agree nor Disagree | 4 Agree | 5  Strongly Agree |
| --- | --- | --- | --- | --- |
|  |  |  |  |  |

3. The job provides me with significant autonomy in making decisions.

| 1  Strongly Disagree | 2 Disagree | 3  Neither Agree nor Disagree | 4 Agree | 5  Strongly Agree |
| --- | --- | --- | --- | --- |
|  |  |  |  |  |

Work Methods Autonomy

1. The job allows me to make decisions about what methods I use to complete my work.

| 1  Strongly Disagree | 2 Disagree | 3  Neither Agree nor Disagree | 4 Agree | 5  Strongly Agree |
| --- | --- | --- | --- | --- |
|  |  |  |  |  |

2. The job gives me considerable opportunity for independence and freedom in how I do the work.

| 1  Strongly Disagree | 2 Disagree | 3  Neither Agree nor Disagree | 4 Agree | 5  Strongly Agree |
| --- | --- | --- | --- | --- |
|  |  |  |  |  |

3. The job allows me to decide on my own how to go about doing my work

| 1  Strongly Disagree | 2 Disagree | 3  Neither Agree nor Disagree | 4 Agree | 5  Strongly Agree |
| --- | --- | --- | --- | --- |
|  |  |  |  |  |

**Task Variety**

1. The job involves a great deal of task variety.

| 1  Strongly Disagree | 2 Disagree | 3  Neither Agree nor Disagree | 4 Agree | 5  Strongly Agree |
| --- | --- | --- | --- | --- |
|  |  |  |  |  |

2. The job involves doing a number of different things.

| 1  Strongly Disagree | 2 Disagree | 3  Neither Agree nor Disagree | 4 Agree | 5  Strongly Agree |
| --- | --- | --- | --- | --- |
|  |  |  |  |  |

3. The job requires the performance of a wide range of tasks. 4. The job involves performing a variety of tasks.

| 1  Strongly Disagree | 2 Disagree | 3  Neither Agree nor Disagree | 4 Agree | 5  Strongly Agree |
| --- | --- | --- | --- | --- |
|  |  |  |  |  |

**Task Significance**

1. The results of my work are likely to significantly affect the lives of other people.

| 1  Strongly Disagree | 2 Disagree | 3  Neither Agree nor Disagree | 4 Agree | 5  Strongly Agree |
| --- | --- | --- | --- | --- |
|  |  |  |  |  |

2. The job itself is very significant and important in the broader scheme of things.

| 1  Strongly Disagree | 2 Disagree | 3  Neither Agree nor Disagree | 4 Agree | 5  Strongly Agree |
| --- | --- | --- | --- | --- |
|  |  |  |  |  |

3. The job has a large impact on people outside the organization.

| 1  Strongly Disagree | 2 Disagree | 3  Neither Agree nor Disagree | 4 Agree | 5  Strongly Agree |
| --- | --- | --- | --- | --- |
|  |  |  |  |  |

4. The work performed on the job has a significant impact on people outside the organization.

| 1  Strongly Disagree | 2 Disagree | 3  Neither Agree nor Disagree | 4 Agree | 5  Strongly Agree |
| --- | --- | --- | --- | --- |
|  |  |  |  |  |

**Task Identity**

1. The job involves completing a piece of work that has an obvious beginning and end.

| 1  Strongly Disagree | 2 Disagree | 3  Neither Agree nor Disagree | 4 Agree | 5  Strongly Agree |
| --- | --- | --- | --- | --- |
|  |  |  |  |  |

2. The job is arranged so that I can do an entire piece of work from beginning to end.

| 1  Strongly Disagree | 2 Disagree | 3  Neither Agree nor Disagree | 4 Agree | 5  Strongly Agree |
| --- | --- | --- | --- | --- |
|  |  |  |  |  |

3. The job provides me the chance to completely finish the pieces of work I begin.

| 1  Strongly Disagree | 2 Disagree | 3  Neither Agree nor Disagree | 4 Agree | 5  Strongly Agree |
| --- | --- | --- | --- | --- |
|  |  |  |  |  |

4. The job allows me to complete work I start.

| 1  Strongly Disagree | 2 Disagree | 3  Neither Agree nor Disagree | 4 Agree | 5  Strongly Agree |
| --- | --- | --- | --- | --- |
|  |  |  |  |  |

**Feedback From Job**

1. The work activities themselves provide direct and clear information about the effectiveness (e.g., quality and quantity) of my job performance.

| 1  Strongly Disagree | 2 Disagree | 3  Neither Agree nor Disagree | 4 Agree | 5  Strongly Agree |
| --- | --- | --- | --- | --- |
|  |  |  |  |  |

2. The job itself provides feedback on my performance.

| 1  Strongly Disagree | 2 Disagree | 3  Neither Agree nor Disagree | 4 Agree | 5  Strongly Agree |
| --- | --- | --- | --- | --- |
|  |  |  |  |  |

3. The job itself provides me with information about my performance.

| 1  Strongly Disagree | 2 Disagree | 3  Neither Agree nor Disagree | 4 Agree | 5  Strongly Agree |
| --- | --- | --- | --- | --- |
|  |  |  |  |  |

**Part 2 - Knowledge Characteristics**

**Job Complexity**

1. The job requires that I only do one task or activity at a time (reverse scored).

| 1  Strongly Agree | 2  Agree | 3  Neither Agree nor Disagree | 4 Disagree | 5  Strongly  Disagree |
| --- | --- | --- | --- | --- |
|  |  |  |  |  |

2. The tasks on the job are simple and uncomplicated (reverse scored).

| 1  Strongly Agree | 2  Agree | 3  Neither Agree nor Disagree | 4 Disagree | 5  Strongly  Disagree |
| --- | --- | --- | --- | --- |
|  |  |  |  |  |

3. The job comprises relatively uncomplicated tasks (reverse scored).

| 1  Strongly Agree | 2  Agree | 3  Neither Agree nor Disagree | 4 Disagree | 5  Strongly  Disagree |
| --- | --- | --- | --- | --- |
|  |  |  |  |  |

4. The job involves performing relatively simple tasks (reverse scored).

| 1  Strongly Agree | 2  Agree | 3  Neither Agree nor Disagree | 4 Disagree | 5  Strongly  Disagree |
| --- | --- | --- | --- | --- |
|  |  |  |  |  |

**Information Processing**

1. The job requires me to monitor a great deal of information.

| 1  Strongly Disagree | 2 Disagree | 3  Neither Agree nor Disagree | 4 Agree | 5  Strongly Agree |
| --- | --- | --- | --- | --- |
|  |  |  |  |  |

2. The job requires that I engage in a large amount of thinking.

| 1  Strongly Disagree | 2 Disagree | 3  Neither Agree nor Disagree | 4 Agree | 5  Strongly Agree |
| --- | --- | --- | --- | --- |
|  |  |  |  |  |

3. The job requires me to keep track of more than one thing at a time.

| 1  Strongly Disagree | 2 Disagree | 3  Neither Agree nor Disagree | 4 Agree | 5  Strongly Agree |
| --- | --- | --- | --- | --- |
|  |  |  |  |  |

4. The job requires me to analyse a lot of information.

| 1  Strongly Disagree | 2 Disagree | 3  Neither Agree nor Disagree | 4 Agree | 5  Strongly Agree |
| --- | --- | --- | --- | --- |
|  |  |  |  |  |

**Problem Solving**

1. The job involves solving problems that have no obvious correct answer.

| 1  Strongly Disagree | 2 Disagree | 3  Neither Agree nor Disagree | 4 Agree | 5  Strongly Agree |
| --- | --- | --- | --- | --- |
|  |  |  |  |  |

2. The job requires me to be creative.

| 1  Strongly Disagree | 2 Disagree | 3  Neither Agree nor Disagree | 4 Agree | 5  Strongly Agree |
| --- | --- | --- | --- | --- |
|  |  |  |  |  |

3. The job often involves dealing with problems that I have not met before.

| 1  Strongly Disagree | 2 Disagree | 3  Neither Agree nor Disagree | 4 Agree | 5  Strongly Agree |
| --- | --- | --- | --- | --- |
|  |  |  |  |  |

4. The job requires unique ideas or solutions to problems.

| 1  Strongly Disagree | 2 Disagree | 3  Neither Agree nor Disagree | 4 Agree | 5  Strongly Agree |
| --- | --- | --- | --- | --- |
|  |  |  |  |  |

**Skill Variety**

1. The job requires a variety of skills.

| 1  Strongly Disagree | 2 Disagree | 3  Neither Agree nor Disagree | 4 Agree | 5  Strongly Agree |
| --- | --- | --- | --- | --- |
|  |  |  |  |  |

2. The job requires me to utilize a variety of different skills in order to complete the work.

| 1  Strongly Disagree | 2 Disagree | 3  Neither Agree nor Disagree | 4 Agree | 5  Strongly Agree |
| --- | --- | --- | --- | --- |
|  |  |  |  |  |

3. The job requires me to use a number of complex or high-level skills.

| 1  Strongly Disagree | 2 Disagree | 3  Neither Agree nor Disagree | 4 Agree | 5  Strongly Agree |
| --- | --- | --- | --- | --- |
|  |  |  |  |  |

4. The job requires the use of a number of skills.

| 1  Strongly Disagree | 2 Disagree | 3  Neither Agree nor Disagree | 4 Agree | 5  Strongly Agree |
| --- | --- | --- | --- | --- |
|  |  |  |  |  |

**Specialization**

1. The job is highly specialized in terms of purpose, tasks, or activities.

| 1  Strongly Disagree | 2 Disagree | 3  Neither Agree nor Disagree | 4 Agree | 5  Strongly Agree |
| --- | --- | --- | --- | --- |
|  |  |  |  |  |

2. The tools, procedures, materials, and so forth used on this job are highly specialized in terms of purpose.

| 1  Strongly Disagree | 2 Disagree | 3  Neither Agree nor Disagree | 4 Agree | 5  Strongly Agree |
| --- | --- | --- | --- | --- |
|  |  |  |  |  |

3. The job requires very specialized knowledge and skills.

| 1  Strongly Disagree | 2 Disagree | 3  Neither Agree nor Disagree | 4 Agree | 5  Strongly Agree |
| --- | --- | --- | --- | --- |
|  |  |  |  |  |

4. The job requires a depth of knowledge and expertise.

| 1  Strongly Disagree | 2 Disagree | 3  Neither Agree nor Disagree | 4 Agree | 5  Strongly Agree |
| --- | --- | --- | --- | --- |
|  |  |  |  |  |

**Part 3 - Social Characteristics**

**Social Support**

1. I have the opportunity to develop close friendships in my job.

| 1  Strongly Disagree | 2 Disagree | 3  Neither Agree nor Disagree | 4 Agree | 5  Strongly Agree |
| --- | --- | --- | --- | --- |
|  |  |  |  |  |

2. I have the chance in my job to get to know other people.

| 1  Strongly Disagree | 2 Disagree | 3  Neither Agree nor Disagree | 4 Agree | 5  Strongly Agree |
| --- | --- | --- | --- | --- |
|  |  |  |  |  |

3. I have the opportunity to meet with others in my work.

| 1  Strongly Disagree | 2 Disagree | 3  Neither Agree nor Disagree | 4 Agree | 5  Strongly Agree |
| --- | --- | --- | --- | --- |
|  |  |  |  |  |

4. My supervisor is concerned about the welfare of the people that work for him/her.

| 1  Strongly Disagree | 2 Disagree | 3  Neither Agree nor Disagree | 4 Agree | 5  Strongly Agree |
| --- | --- | --- | --- | --- |
|  |  |  |  |  |

5. People I work with take a personal interest in me.

| 1  Strongly Disagree | 2 Disagree | 3  Neither Agree nor Disagree | 4 Agree | 5  Strongly Agree |
| --- | --- | --- | --- | --- |
|  |  |  |  |  |

6. People I work with are friendly.

| 1  Strongly Disagree | 2 Disagree | 3  Neither Agree nor Disagree | 4 Agree | 5  Strongly Agree |
| --- | --- | --- | --- | --- |
|  |  |  |  |  |

**Interdependence**

Initiated Interdependence

1. The job requires me to accomplish my job before others complete their job.

| 1  Strongly Disagree | 2 Disagree | 3  Neither Agree nor Disagree | 4 Agree | 5  Strongly Agree |
| --- | --- | --- | --- | --- |
|  |  |  |  |  |

2. Other jobs depend directly on my job.

| 1  Strongly Disagree | 2 Disagree | 3  Neither Agree nor Disagree | 4 Agree | 5  Strongly Agree |
| --- | --- | --- | --- | --- |
|  |  |  |  |  |

3. Unless my job gets done, other jobs cannot be completed.

| 1  Strongly Disagree | 2 Disagree | 3  Neither Agree nor Disagree | 4 Agree | 5  Strongly Agree |
| --- | --- | --- | --- | --- |
|  |  |  |  |  |

Received Interdependence

1. The job activities are greatly affected by the work of other people.

| 1  Strongly Disagree | 2 Disagree | 3  Neither Agree nor Disagree | 4 Agree | 5  Strongly Agree |
| --- | --- | --- | --- | --- |
|  |  |  |  |  |

2. The job depends on the work of many different people for its completion.

| 1  Strongly Disagree | 2 Disagree | 3  Neither Agree nor Disagree | 4 Agree | 5  Strongly Agree |
| --- | --- | --- | --- | --- |
|  |  |  |  |  |

3. My job cannot be done unless others do their work.

| 1  Strongly Disagree | 2 Disagree | 3  Neither Agree nor Disagree | 4 Agree | 5  Strongly Agree |
| --- | --- | --- | --- | --- |
|  |  |  |  |  |

Interaction Outside Organization

1. The job requires spending a great deal of time with people outside my organization.

| 1  Strongly Disagree | 2 Disagree | 3  Neither Agree nor Disagree | 4 Agree | 5  Strongly Agree |
| --- | --- | --- | --- | --- |
|  |  |  |  |  |

1. The job involves interaction with people who are not members of my organization.

| 1  Strongly Disagree | 2 Disagree | 3  Neither Agree nor Disagree | 4 Agree | 5  Strongly Agree |
| --- | --- | --- | --- | --- |
|  |  |  |  |  |

3. On the job, I frequently communicate with people who do not work for the same organization as I do.

| 1  Strongly Disagree | 2 Disagree | 3  Neither Agree nor Disagree | 4 Agree | 5  Strongly Agree |
| --- | --- | --- | --- | --- |
|  |  |  |  |  |

4. The job involves a great deal of interaction with people outside my organization.

| 1  Strongly Disagree | 2 Disagree | 3  Neither Agree nor Disagree | 4 Agree | 5  Strongly Agree |
| --- | --- | --- | --- | --- |
|  |  |  |  |  |

**Feedback From Others**

1. I receive a great deal of information from my manager and co-workers about my job performance.

| 1  Strongly Disagree | 2 Disagree | 3  Neither Agree nor Disagree | 4 Agree | 5  Strongly Agree |
| --- | --- | --- | --- | --- |
|  |  |  |  |  |

2. Other people in the organization, such as managers and co-workers, provide information about the effectiveness (e.g., quality and quantity) of my job performance.

| 1  Strongly Disagree | 2 Disagree | 3  Neither Agree nor Disagree | 4 Agree | 5  Strongly Agree |
| --- | --- | --- | --- | --- |
|  |  |  |  |  |

3. I receive feedback on my performance from other people in my organization (such as my manager or co-workers).

| 1  Strongly Disagree | 2 Disagree | 3  Neither Agree nor Disagree | 4 Agree | 5  Strongly Agree |
| --- | --- | --- | --- | --- |
|  |  |  |  |  |

**Part 4 - Work Context**

**Ergonomics**

1. The seating arrangements on the job are adequate (e.g., ample opportunities to sit, comfortable chairs, good postural support).

| 1  Strongly Disagree | 2 Disagree | 3  Neither Agree nor Disagree | 4 Agree | 5  Strongly Agree |
| --- | --- | --- | --- | --- |
|  |  |  |  |  |

2. The work place allows for all size differences between people in terms of clearance, reach, eye height, leg room, etc.

| 1  Strongly Disagree | 2 Disagree | 3  Neither Agree nor Disagree | 4 Agree | 5  Strongly Agree |
| --- | --- | --- | --- | --- |
|  |  |  |  |  |

3. The job involves excessive reaching (reverse scored).

| 1  Strongly Agree | 2  Agree | 3  Neither Agree nor Disagree | 4 Disagree | 5  Strongly  Disagree |
| --- | --- | --- | --- | --- |
|  |  |  |  |  |

**Physical Demands**

1. The job requires a great deal of muscular endurance.

| 1  Strongly Disagree | 2 Disagree | 3  Neither Agree nor Disagree | 4 Agree | 5  Strongly Agree |
| --- | --- | --- | --- | --- |
|  |  |  |  |  |

2. The job requires a great deal of muscular strength.

| 1  Strongly Disagree | 2 Disagree | 3  Neither Agree nor Disagree | 4 Agree | 5  Strongly Agree |
| --- | --- | --- | --- | --- |
|  |  |  |  |  |

3. The job requires a lot of physical effort.

| 1  Strongly Disagree | 2 Disagree | 3  Neither Agree nor Disagree | 4 Agree | 5  Strongly Agree |
| --- | --- | --- | --- | --- |
|  |  |  |  |  |

**Work Conditions**

1. The work place is free from excessive noise.

| 1  Strongly Disagree | 2 Disagree | 3  Neither Agree nor Disagree | 4 Agree | 5  Strongly Agree |
| --- | --- | --- | --- | --- |
|  |  |  |  |  |

2. The climate at the work place is comfortable in terms of temperature and humidity.

| 1  Strongly Disagree | 2 Disagree | 3  Neither Agree nor Disagree | 4 Agree | 5  Strongly Agree |
| --- | --- | --- | --- | --- |
|  |  |  |  |  |

3. The job has a low risk of accident.

| 1  Strongly Disagree | 2 Disagree | 3  Neither Agree nor Disagree | 4 Agree | 5  Strongly Agree |
| --- | --- | --- | --- | --- |
|  |  |  |  |  |

4. The job takes place in an environment free from health hazards (e.g., chemicals, fumes, etc.).

| 1  Strongly Disagree | 2 Disagree | 3  Neither Agree nor Disagree | 4 Agree | 5  Strongly Agree |
| --- | --- | --- | --- | --- |
|  |  |  |  |  |

5. The job occurs in a clean environment.

| 1  Strongly Disagree | 2 Disagree | 3  Neither Agree nor Disagree | 4 Agree | 5  Strongly Agree |
| --- | --- | --- | --- | --- |
|  |  |  |  |  |

**Equipment Use**

1. The job involves the use of a variety of different equipment.

| 1  Strongly Disagree | 2 Disagree | 3  Neither Agree nor Disagree | 4 Agree | 5  Strongly Agree |
| --- | --- | --- | --- | --- |
|  |  |  |  |  |

2. The job involves the use of complex equipment or technology.

| 1  Strongly Disagree | 2 Disagree | 3  Neither Agree nor Disagree | 4 Agree | 5  Strongly Agree |
| --- | --- | --- | --- | --- |
|  |  |  |  |  |

3. A lot of time was required to learn the equipment used on the job.

| 1  Strongly Disagree | 2 Disagree | 3  Neither Agree nor Disagree | 4 Agree | 5  Strongly Agree |
| --- | --- | --- | --- | --- |
|  |  |  |  |  |

1. Morgeson FP, Humphrey SE. The Work Design Questionnaire (WDQ): developing and validating a comprehensive measure for assessing job design and the nature of work. J Appl Psychol. 2006;91(6):1321-39.
